# Burnout in Dentists and the COVID-19 Pandemic: A Systematic Review

**DOI:** 10.2174/0117450179400081250815115140

**Published:** 2025-08-19

**Authors:** Juliane Kely Fagundes Silva, Liliane Lins-Kusterer, Marcela Beatriz Aguiar Moreira, Fernando Martins Carvalho

**Affiliations:** 1 Postgraduate Programme in Collective Health, Federal University of Bahia, Salvador, Brazil; 2 School of Medicine of Bahia, Federal University of Bahia, Salvador, Brazil; 3 Postgraduate Programme in Collective Health, Feira de Santana State University, Feira de Santana, Brazil

**Keywords:** Dentists, Professional burnout, Maslach burnout inventory, COVID-19, Emotional exhaustion, Depersonalization

## Abstract

**Introduction:**

This study aimed to identify and analyze research on burnout in dentists, measured both prior to and during the COVID-19 pandemic, using the Maslach Burnout Inventory (MBI).

**Methods:**

A systematic literature review was conducted across five databases using the search terms “Dentists” and “Burnout, Psychological.” Articles published between 1981 and December 2024 that utilized the MBI were included. Studies were classified based on the time of data collection: either prior to or during the COVID-19 pandemic (defined as January 30, 2020, to May 5, 2023).

**Results:**

We selected 15 of the 1,486 articles identified. Eleven of these reported means and standard deviations for the burnout scales. Among them, eight calculated scale means and standard deviations according to the guidelines recommended in the MBI manual; six studies were conducted prior to the pandemic, and two during it. An initial analysis suggests that mean levels of Emotional Exhaustion and Depersonalization increased during the pandemic, while mean levels of Personal Accomplishment remained comparable to pre-pandemic levels. However, five studies used different cutoff points to define low, moderate, or high burnout levels for each scale, limiting comparability across studies.

**Discussion:**

Few articles have adequately utilized the MBI to assess burnout in dental surgeons either before or during the COVID-19 pandemic.

**Conclusion:**

Theoretical arguments suggest that the COVID-19 pandemic may have adversely affected burnout levels in dentists. However, the studies we analyzed offer only limited evidence supporting an increase in the burnout dimensions of Emotional Exhaustion and Depersonalization during the pandemic.

## 1 INTRODUCTION

Burnout syndrome results from chronic workplace stress that has not been successfully managed [1]. It is characterized by three dimensions: emotional exhaustion (EE), which involves feelings of depletion of energy or exhaustion; depersonalization (DP), marked by mental detachment from one’s work or negative, cynical attitudes toward it; and low personal accomplishment (PA), reflecting feelings of professional ineffectiveness [2]. Factors contributing to burnout in dentists include unfavorable working conditions, such as limited appointment times, an exhausting workload, working without assistants, isolation from the health team, shortages of essential materials for workplace safety, including personal protective equipment, an inadequate physical structure of the health facility, and fragile employment relationships [3]. Burnout also arises from the nature of dental work, which exposes dentists to various stressors, including noise, the risk of contamination by pathogenic agents in body fluids and chemical substances, exposure to radiation, physical demands related to ergonomics, anxiety experienced by dental patients, and conflicting interpersonal relationships at work [4, 5].

The literature on burnout in oral health professionals is extensive, yet gaps remain regarding how to consistently track and assess burnout according to the guidelines of validated instruments. When using the Maslach Burnout Inventory (MBI) questionnaire, some researchers adopt cut-off points to classify each of the three burnout dimensions as low, moderate, or high; others treat the data from these dimensions as continuous variables (averages). Still, others use a dichotomous approach (presence or absence of burnout) from a diagnostic perspective. However, the developers of the MBI do not recommend using statistical cut-off scores to identify individuals with burnout, as these lack diagnostic validity [6].

In recent years, the COVID-19 pandemic has brought renewed attention to the worsening of burnout and other work-related mental health conditions across healthcare and many other sectors. The World Health Organization declared COVID-19 a Public Health Emergency of International Concern on January 30, 2020, and announced its end on May 5, 2023 [7].

Thus, the aim of this systematic review was to identify studies on burnout in dentists, as measured prior to and during the COVID-19 pandemic, using the Maslach Burnout Inventory.

## 2 METHODS

### 2.1 Study Design

This systematic review was registered with the International Prospective Register of Systematic Reviews (PROSPERO) under number CRD42024620831. The scientific writing was based on the Preferred Reporting Items for Systematic Reviews and Meta-Analyses: The PRISMA-P Statement [8]. We used the PECO strategy, considering the following: Population – dentists; Exposure – burnout; Comparison – studies conducted before and during the COVID-19 pandemic; and Outcome – the three dimensions of burnout syndrome [9].

### 2.2 Eligibility Criteria

The following inclusion criteria were applied: cross-sectional, case-control, and cohort epidemiological studies; a study population of dentists with burnout as the outcome; use of the Maslach Burnout Inventory (MBI) to assess the three burnout scales (emotional exhaustion, depersonalization, and low personal accomplishment); and articles published between 1981 (the year the MBI was published) and December 2024. The following were excluded: review studies, case reports, and letters to the editor; studies investigating the same population as a selected article; studies that did not present a global average for each scale or did not mention cutoff points for categorizing each burnout dimension; and those that presented a dichotomous perspective (has/does not have burnout), a diagnosis of “burnout cases,” or used the term “general exhaustion.” There were no restrictions on the language of publication. Only full-text articles were included in the search.

### 2.3 Data Source and Search Strategy

The studies were accessed in December 2024 from the following electronic databases: Medical Literature Analysis and Retrieval System Online (Pubmed/MEDLINE), Web of Science, Scopus, Embase, Biblioteca Virtual em Saúde (BVS), and PsycINFO. The search used the following terms: “Dentists” and “Burnout, Psychological”, as well as their synonyms, as identified in the Medical Subject Headings (MeSH). The Boolean operator “AND” was added to these descriptors. These strategies were subjected to validation through the “Peer Review of Electronic Search Strategies” (PRESS) guidelines for systematic reviews [10].

### 2.4 Study Selection

The article selection process was carried out independently by two reviewers, without prior knowledge of the authors’ evaluations. The studies were selected through independent reading of titles and abstracts by two researchers. In cases of disagreement regarding the inclusion or exclusion of an article, consensus was reached through discussion. The selected articles were then read completely.

### 2.5 Assessment of the Methodological Quality of the Articles

The methodological quality of the selected studies was assessed using the Newcastle-Ottawa Quality Assessment Scale for case-control and cohort studies [11]; for cross-sectional studies, a modified version of this scale was employed [12]. Only articles scoring seven or higher were included in this review [13].

### 2.6 Statistical Analysis

The articles were classified according to their time of data collection, whether before (initiation of data collection up to January 29, 2020) or during (from January 30, 2020 to May 5, 2023) the COVID-19 pandemic. The MBI scores, expressed as means or proportions, were, therefore, stratified according to the timing of the pandemic.

## 3 RESULTS

### 3.1 Selected Studies

**Fig. (1):**
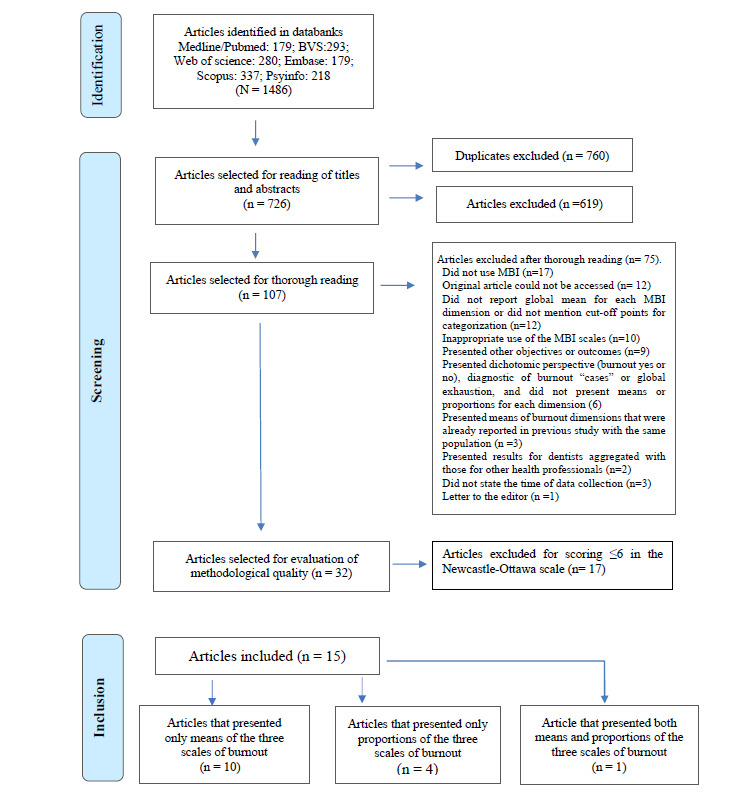
Flowchart of the search and selection processes of the studies included in this review.

After removing 760 duplicates, 726 of the 1,486 identified articles remained, of which 616 were excluded following a reading of the titles and abstracts. One hundred and seven articles were therefore selected for full reading; of these, 75 were excluded for various reasons. The main justifications for excluding these articles were the non-use of the MBI (n = 17) and difficulty in accessing the text (n = 12), followed by the absence of a global average for each scale or cutoff point to categorize the burnout scales (n = 12). Inadequate use of the MBI was also a notable justification (n=10). Of the 32 articles evaluated by the Newcastle Ottawa scale, 15 studies met all the inclusion criteria (Fig. **1**).

### 3.2 Characteristics of the Selected Studies

All 15 selected studies were cross-sectional and were published between 1998 and 2024. Ten articles contained data collected prior to the COVID-19 pandemic, while five collected data during the health crisis. No article included data collected after the end of the Public Health Emergency of International Concern related to COVID-19. Although one article [14] contained data collected between March and June 2023, it did not analyze burnout data related to COVID-19. The 15 studies were conducted in 13 countries, with Brazil and the Netherlands contributing two studies each. One article [15] assessed both proportions and means.

### 3.3 Prevalence of Burnout According to Cut-off Points

Five studies [14-18] reported the proportion of individuals affected by the three burnout scales. The cut-off points for defining Low, Moderate, or High levels in each burnout scale varied widely between the studies. Thus, the Emotional Exhaustion scale had cut-off points ranging from ≤13 to ≤18 for Low; from 14-22 to 19-26 for Moderate; and from ≥23 to ≥27 for High level EE. The Depersonalization scale had cut-off points ranging from ≤2 to ≤6 for Low, from 3-5 to 7-12 for Moderate, and from ≥6 to ≥13 for High-level DP. The Personal Accomplishment scale had cutoff points ranging from ≥39 to ≥40 for Low; from 32-38 to 34-39 for Moderate; and from ≤31 to ≤33 for High level PA. Only three studies [14-16] used the same cutoff points to define the proportions for each scale. Of the five studies that evaluated the proportions of the three burnout scales, only one [17] collected data prior to the COVID-19 pandemic (before January 29, 2020). The proportions of high EE, high DP, and low PA were 20%, 29%, and 65%, respectively. Of the four studies that identified the proportions of the burnout scales during the COVID-19 pandemic, the prevalence of high EE ranged from 19.2% to 62%; for high DP, this ranged from 9.3% to 36%, while for low PA, the prevalence ranged from 6.6% to 52% Table **1**.

### 3.4 Means of Burnout Scales

Eleven studies [5, 15, 19-27] presented the results of each burnout scale as means and standard deviations. Two articles [19-21] used a Dutch version of the MBI containing 20 questions, excluding questions 12 and 16. A third study [23] utilized a version of the MBI-General Survey that consisted of 16 questions and was translated into Brazilian Portuguese (Table **2**). Disregarding the three studies mentioned above, eight articles remained, of which six [5, 20, 23-26] contained data collected prior to the pandemic and two [17, 27] contained data collected during it. There were overlaps in the ranges of variation for the means and respective standard deviations of each of the three burnout scales, comparing the periods prior to and during the pandemic. Thus, EE ranged from 11.5 to 30.8 (before) to 25.68 to 28.9 (after); DP from 2.3 to 11.22 (before) to 8.2 to 8.61 (during); while PA ranged from 34.4 to 43.8 (before) to 28.0 to 35.31 (during) (Table **2**).

## 4 DISCUSSION

### 4.1 The COVID-19 Pandemic and Burnout in Dentists

The results of this review revealed a similar pattern in the range of mean scores for the burnout scales, regardless of whether data collection occurred before or during the pandemic. The study by Ahmad *et al.* [15] aimed to identify the prevalence of burnout in dentists in Pakistan during the COVID-19 pandemic. Although the mean values for Emotional Exhaustion (EE), Depersonalization (DP), and Personal Accomplishment (PA) were considered moderate, 47.5% of respondents exhibited high levels of EE, and 29.4% demonstrated low levels of PA. The authors highlighted the lack of pre-pandemic studies in the country, which hindered the assessment of the pandemic’s impact on burnout levels in dentists.

In the study by Silva *et al.* [27] involving Brazilian dentists during the COVID-19 pandemic, the mean burnout scores were: EE = 28.9 ± 8.9; DP = 8.2 ± 6.3; and PA = 28.0 ± 6.2. These values are higher for EE and DP and lower for PA compared with the normative mean values suggested by the MBI-HSS manual [6]: EE = 21.35 (10.51); DP = 7.46 (5.11); PA = 32.75 (7.71).

In this review, it was not possible to perform a meta-analysis on the proportions of the three MBI scales, since the cutoff points differed greatly between the studies. The meta-analysis of the means of the burnout dimensions was also not feasible, since only two articles conducted during the pandemic presented these data. Moro *et al.* [28] conducted a meta-analysis of the literature on burnout in dentists (DSs) who used the MBI, covering publications from 1989 to 2020. However, none of the included articles explored the association between burnout and the COVID-19 pandemic. This meta-analysis, based on fifteen studies, estimated the prevalence of high Emotional Exhaustion (EE) at 25%, high Depersonalization (DP) at 18%, and low Personal Accomplishment (PA) at 32%. It is essential to note that this review did not consider the possibility that the 15 studies may have employed different cutoff points to classify the scores of each scale as Low, Moderate, or High. The meta-analysis also estimated the mean scores for each burnout scale based on 14 studies: EE = 17.90; DP = 6.93; and PA = 34.69. Considering the normative mean levels suggested by the MBI-HSS manual [6], it can be concluded that the studies reported by Moro *et al.* [28] indicated lower mean values for EE and DP and a slightly higher mean value for PA.

We also observed higher mean values for EE and DP, and similar mean values for PA, when comparing the results of the two studies conducted during the pandemic [15, 27] with the mean values reported by Moro *et al.* [28].

### 4.2 Inappropriate uses of MBI Scores

We identified inappropriate use of the MBI in the literature we investigated. The first point of concern is that the MBI-HSS manual [6] recommends assessing the three burnout dimensions as continuous variables, presenting mean and standard deviation values for each. The developers of the MBI-HSS do not support the use of cutoff points to classify burnout as low, moderate, or high, considering this practice arbitrary and lacking diagnostic validity [29]. However, many studies, such as those presented in Table **1**, apply cutoff points to categorize data, presenting results in terms of proportions. Additionally, we found studies that created “macro-categories” such as “high burnout” (characterized by high EE and DP scores and low PA scores), “low burnout” (low EE and DP scores and high PA scores), and “moderate burnout” for other combinations [23, 26]. This methodological diversity hinders comparability between studies and raises concerns about the reliability of the results.

## CONCLUSION

This review identified only two studies on burnout in dentists that were conducted during the COVID-19 pandemic and used the Maslach Burnout Inventory. The heterogeneity of the reviewed studies, particularly the use of different cutoff points to define burnout levels in the three MBI dimensions, hinders direct comparisons. Although theoretical arguments support the hypothesis that the pandemic may have negatively impacted burnout levels in this population, the findings of this systematic review do not allow us to categorically conclude that the pandemic led to a worsening in the mean values of the three MBI dimensions.

However, an initial analysis suggests that mean levels of the Emotional Exhaustion and Depersonalization scales have increased, while mean levels of the Personal Accomplishment scale remain comparable to those reported prior to the pandemic.

## Figures and Tables

**Table 1:** Five Studies about burnout in dentists that presented cut-off points and proportion of affected individuals for the MBI three scales.

**Authors, Publication year [reference]**	**Time of data collection,** **Country**	**Number of dentists**	**Cut-off points for categorization**	**Proportion in the three burnout scales**
Badrasawi *et al.*, 2024 [14]	During the pandemic, Palestine	271	EE: Low: ≤ 16 Moderate: 17-26 Alto: ≥ 27 DP: Low: ≤ 6 Moderate: 7-12 High: ≥ 13 PA: Low:≥ 39 Moderate: 32 -38 High: ≤ 31	EE: Low: 21% Moderate: 31% High: 48% DP: Low: 60.5% Moderate: 25.1% High: 14.4% PA: Low: 52% Moderate: 26.9% High: 21%
Ahmad *et al.*, 2023 [15]	During the pandemic, Pakistan	282	EE: Low: ≤ 16 Moderate: 17-26 High: ≥ 27 DP: Low: ≤ 6 Moderate: 7-12 High: ≥ 13 RP: Low:≥ 39 Moderate: 32 -38 High: ≤ 31	EE: Low: 19.9% Moderate: 32.6% High: 47.5% DP: Low: 44% Moderate: 33% High: 23% PA: Low: 29.4% Moderate: 26.2% High: 44.3%
Radwan and Morsy, 2022 [16]	During the pandemic, Egypt	100	EE: Low: ≤ 16 Moderate: 17-26 High: ≥ 27 DP: Low: ≤ 6 Moderate: 7-12 High: ≥ 13 PA: Low: ≥ 39 Moderate: 32 -38 High: ≤ 31	EE: Low: 17% Moderate: 21% High: 62% DP: Low: 43% Moderate: 21% High: 36% PA: Low: 23% Moderate: 31% High: 46%
Pirillo, Caracciolo and Siciliani, 2011 [17]	Before the pandemic, Italy	366	EE: Low: ≤ 13 Moderate: 14-22 High: ≥ 23 DP: Low: ≤ 2 Moderate: 3-5 High: ≥ 6 PA: Low: ≥ 39 Moderate: 32-38 High: ≤ 31	EE: Low: 55% Moderate: 25% High: 20% DP: Low: 51% Moderate: 20% High: 29% PA: Low: 65% Moderate: 24% High: 11%
Verástegui-Sandoval *et al.*, 2024 [18]	During the pandemic, Peru	182	EE Low: ≤ 18 Moderate: 19-26 High: ≥ 27 DP: Low: ≤ 5 Moderate: 6-9 High: ≥ 10 PA: Low: ≥ 40 Moderate: 34-39 High: ≤ 33	EE: Low: 70.9% Moderate: 9.9% High: 19.2% DP: Low: 76.4% Moderate: 14.3% High: 9.3% PA: Low: 6.6% Moderate: 11% High: 82.4%

**Table 2:** Eleven studies about burnout in dentists that presented the mean **and standard deviation for the MBI three scales.**

**Authors, year** **[reference]**	**Time of data collection,** **Country**	**Number of dentists**	**Mean and standard deviation of the burnout scales**
Gorter *et al.*, 1998 [19]	Before the pandemic, Holland	709	EE: 13.7 (8.6) DP: 5.9 (3.9) PA: 30.8 (5.9)
Divaris *et al.*, 2012 [20]	Before the pandemic, Switzerland	43	EE: 14.3 (9.5) DP: 3.7 (4.2) PA: 34.4 (7.6)
Gorter, Jacobs and Allard, 2012 [21]	Before the pandemic, Holland	110	EE: 1.18 (0.90) DP: 0.85 (0.63) PA: 4.89 (0.72)
Porto *et al.*, 2014 [22]	Before the pandemic, Brazil	116	EE: 12.11(8.26) DP: 2.16 (3.54) PA: 30.56 (5.83)
Jin *et al.*, 2015 [23]	Before the pandemic, South Korea	444	EE: 26.16 (11.4) DP: 11.22 (6.3) PA: 36.54 (8.4)
Calvo *et al.*, 2021 [5]	Before the pandemic, USA	167	EE: 18.0 (11.7)^a^ DP: 6.0 (5.5)^a^ PA: 38.4 (7.2)^a^
Slabsinskiene *et al.*, 2021 [24]	Before the pandemic, Lithuania	380	EE: 24.7 (11.66) DP: 7.8 (5.94) PA: 35.6 (7.66)
Gómez-Polo *et al.*, 2021 [25]	Before the pandemic, Spain	1298	EE: 30.8 (10.9) DP: 10.3 (4.7) PA: 39.8 (5.9)
Hernández *et al.*, 2022 [26]	Before the pandemic, Colombia	117	EE: 11.5 (7.4) DP: 2.3 (3.6) PA: 43.8 (4.6)
Ahmad *et al.*, 2023 [15]	During the pandemic, Pakistan	282	EE: 25.68 (10.2) DP: 8.61 (5.96) PA: 35.31(9.00)
Silva *et al.*, 2023 [27]	During the pandemic, Brazil	251	EE: 28.9 (8.9) DP: 8.2 (6.3) PA: 28.0 (6.2)

^a^ Scale scores were transformed using Method 1 (Sum), instead of Method 2 (Average).

## Data Availability

The data that support the findings of this study are available from the corresponding author (L.L-K.) upon reasonable request.

## LIST OF ABBREVIATIONS

MBI: Maslach Burnout Inventory
MBI-HSS: Maslach Burnout Inventory-Human Services Survey
EE: Emotional exhaustion
DP: Depersonalization
PA: Personal Accomplishment

## STANDARDS OF REPORTING

PRISMA-2020 guidelines and methodology were followed.

## SUPPLEMENTARY MATERIAL

PRISMA checklist is available as supplementary material on the publisher’s website along with the published article.
